# Non-Pharmacological Interventions to Prevent Oropharyngeal Candidiasis in Patients Using Inhaled Corticosteroids: A Narrative Review

**DOI:** 10.3390/healthcare13141718

**Published:** 2025-07-17

**Authors:** Leonardo Arzayus-Patiño, Vicente Benavides-Córdoba

**Affiliations:** 1 Faculty of Health, Physiotherapy Program, Universidad Santiago de Cali, Cali 760035, Colombia; 2 Facultad de Salud, Pharmacology Area, Universidad del Valle, Cali 760032, Colombia; vicente.benavides@correounivalle.edu.co

**Keywords:** oropharyngeal candidiasis, fungal infection, oral hygiene, education, adverse reactions

## Abstract

Inhaled corticosteroids (ICSs) are widely used to manage chronic respiratory conditions such as asthma, chronic obstructive pulmonary disease (COPD), and human immunodeficiency virus (HIV). However, prolonged use of ICS is associated with the development of oropharyngeal candidiasis, a fungal infection primarily caused by *Candida albicans*, due to local immunosuppression in the oral cavity. The incidence of oropharyngeal candidiasis varies depending on geographic region, patient age, and comorbidities, with immunocompromised individuals, those with diabetes, and the elderly being particularly vulnerable. Key risk factors include high ICS doses, poor oral hygiene, and improper use of inhalers. Prevention is the cornerstone of managing oropharyngeal candidiasis associated with the chronic use of inhaled corticosteroids. Patient education on proper inhaler technique and oral hygiene is essential to reduce the risk of fungal overgrowth in the oral cavity. Additional preventive strategies include the use of spacers, mouth rinsing after inhalation, and proper denture care. In cases where these measures fail to prevent the infection, prompt detection and early intervention are crucial to prevent progression or recurrence. This narrative review aims to analyze the most effective prophylactic measures to prevent oropharyngeal candidiasis associated with the chronic use of inhaled corticosteroids, emphasizing patient education, oral hygiene, and proper use of inhalation devices.

## 1. Introduction

Oropharyngeal candidiasis is a common fungal infection primarily caused by the overgrowth of Candida species on the oral mucosa. This condition affects various populations, particularly immunocompromised individuals, patients with prolonged antibiotic use, diabetics, and those using medical devices such as dental prostheses or inhalers.

The prevalence varies depending on the population and risk factors but represents a frequent cause of oral discomfort and can lead to complications if not properly treated [1]. Clinical manifestations include white plaques on the mucosa, redness, and pain, which impact quality of life and, in some cases, predispose to systemic infections. Treatment typically involves a combination of local and systemic measures to eradicate the fungus and prevent recurrences [2].

Among the iatrogenic factors that predispose to oropharyngeal candidiasis, inhaled corticosteroids (ICS) are particularly relevant due to their widespread use in managing chronic respiratory diseases such as asthma and chronic obstructive pulmonary disease (COPD). Despite their clinical benefits, prolonged use of ICS may cause local immunosuppression on the oropharyngeal mucosa, facilitating Candida colonization and proliferation [3].

ICS are synthetic steroid analogs modified to enhance their potency and specificity. Their structure, based on cyclopentanoperhydrophenanthrene, has been altered at key sites such as halogenation and the addition of functional groups to improve receptor affinity, anti-inflammatory activity, and bioavailability in lung tissue [4,5]. These changes result in faster action, longer duration, and effective inflammation control with minimal systemic effects [6].

Exhibit potent glucocorticoid activity, acting at the cellular level to reverse capillary permeability and stabilize lysosomes, thereby reducing inflammation. ICS acts on glucocorticoid receptors (GR) in the cytoplasm. GRα remains in a multiprotein complex that prevents its translocation to the nucleus. Upon binding to the receptor, the steroid-GR complex becomes hyperphosphorylated, dissociates, and moves into the nucleus, where it binds to specific genes, increasing transcription and gene product synthesis [7]. The resulting complex can either activate or repress proteins that initiate the transcription of inflammatory genes, affecting the production of pro-inflammatory cytokines and, consequently, inflammation. Transcription factors such as activator protein 1 (AP-1) and nuclear factor kappa-light-chain-enhancer of activated B cells (NF-κB) are particularly suppressed, especially in asthma. ICSs reverse the activating effect of these factors by recruiting histone deacetylase 2 (HDAC2) to activated inflammatory genes. They can also inhibit MAP kinase signaling pathways. Research is ongoing to find GR ligands with high anti-inflammatory activity and an improved safety profile [4].

By modulating gene transcription, increasing anti-inflammatory genes, and suppressing inflammatory ones, ICS are effective in asthma. This occurs by reducing TH2 cell cytokines and inducing eosinophil apoptosis, improving lung function by decreasing chronic inflammation and hyperreactivity [8]. According to the GINA guidelines, ICSs prevent exacerbations and improve the quality of life in asthma patients, with treatments adjusted according to severity [9].

In chronic obstructive pulmonary disease (COPD), ICSs provide modest benefits without significantly influencing FEV1 decline or mortality. When combined with a long action beta2 agonist (LABA) or long action muscarinic antagonist (LAMA), they can reduce exacerbations, and triple therapy (ICS/LABA/LAMA) has shown superior efficacy in this regard. However, ICSs use in COPD carries risks such as pneumonia [10]. The GOLD guidelines recommend their use in patients with high blood eosinophil levels (≥300 cells/µL) and discourage their use in those with fewer than 100 cells/µL, emphasizing the need to balance benefits and risks [11,12]. Table 1 presents a classification of commonly used inhaled corticosteroids (ICS), highlighting key features such as duration of action, anti-inflammatory potency, and available formulations.

However, while ICSs have fewer systemic effects compared to oral or parenteral corticosteroids [14,15], they can cause undesirable effects that impact the hypothalamic-pituitary-adrenal (HPA) axis, growth in children, bones, skin, eyes, and immunity. During pregnancy, although ICSs are the preferred option for controlling asthma, they should be used with caution due to potential risks to the fetus. These effects, such as adrenal suppression, are more noticeable with high doses and vary depending on the type of steroid, with fluticasone being more potent than budesonide [16].

Although these adverse reactions are significant, oropharyngeal candidiasis is the most common and, paradoxically, one of the most easily preventable. This condition is considered a consequence of local immunosuppression on the surface of the oral mucosa, and although the intended action of ICS is in the lower airways, this effect occurs secondarily in the deposition of particles in the upper airways [3,4,5,6,7,8,9,10,11,12,13,14,15,16,17]. Prophylaxis is the best strategy to prevent this condition. Through education on proper inhaler use and oral hygiene, the incidence of this condition can be minimized, preventing the progression of the infection that may eventually require pharmacological interventions to avoid complications. This narrative review aims to analyze the most effective prophylactic measures to prevent oropharyngeal candidiasis associated with the chronic use of ICSs, emphasizing the importance of patient education, oral hygiene strategies, proper use of inhalation devices, and pharmacological management when prevention is insufficient.

## 2. Literature Search Strategy

This narrative review was conducted to synthesize the current evidence on non-pharmacological strategies for preventing oropharyngeal candidiasis associated with inhaled corticosteroid (ICS) use. A comprehensive search of the literature was performed using the following electronic databases: PubMed/MEDLINE, Scopus, and Web of Science.

The search strategy combined free-text terms and controlled vocabulary (MeSH terms) related to the main concepts of the review. The following search terms were used:(“Inhaled corticosteroids” OR “ICS”) AND (“oropharyngeal candidiasis” OR “oral candidiasis” OR “thrush” OR “fungal infection” OR “mycoses”) AND (“prevention” OR “non-pharmacological strategies” OR “oral hygiene” OR “mouthwash” OR “spacer devices” OR “education”) AND (“Adverse reactions” OR “Drug-related side effects”).

In PubMed, MeSH terms such as “Candidiasis, Oral”, “Glucocorticoids/administration & dosage”, “Education”, and “Administration, Inhalation” were used to refine the results. Searches were limited to articles published in English or Spanish between January 2000 and March 2025. Additional relevant articles were identified through manual screening of the reference lists of included studies and relevant reviews.

Original research articles, clinical trials, observational studies, systematic reviews, and expert consensus guidelines addressing the prevention of ICS-associated oropharyngeal candidiasis were included. Studies focusing solely on pharmacological treatments of candidiasis or unrelated complications of ICS use were excluded.

The final selection of articles was based on relevance to the topic and quality of the evidence, as judged independently by two reviewers. Discrepancies were resolved by discussion and consensus.

## 3. Secondary Candidiasis Due to ICS

### 3.1. Presentation

Candidiasis, also known as thrush, is an opportunistic fungal infection that frequently manifests in the oral cavity, especially in patients using ICS [18]. This condition is generally caused by *Candida albicans*, a dimorphic yeast that can present as both hyphal and yeast forms depending on the environment. *Candida*, a fungus that is part of the normal oral flora, can proliferate due to ICS use and is isolated in approximately 80% of lesions [19,20]. Although these medications are effective in treating respiratory conditions such as asthma, they can disrupt the microbial balance in the oral cavity, creating an environment conducive to *Candida* overgrowth and, consequently, the development of candidiasis [21].

Although less common, other yeasts can also be isolated, including Candida glabrata (*Nakaseomyces glabratus*), *Candida tropicalis*, *Candida krusei* (*Pichia kudriavzevii*), *Candida guilliermondii* (*Meyerozyma guilliermondii*), *Candida lusitaniae* (*Clavispora lusitaniae*), *Candida parapsilosis*, *Candida pseudotropicalis* (now considered a synonym of *Candida kefyr*), and *Candida stellatoidea* (currently regarded as a variant of Candida albicans). It has been observed that non-albicans Candida species colonize patients aged 80 years or older more frequently than younger patients [22,23].

The clinical presentation of candidiasis secondary to ICS use is characterized by the appearance of white, cottony plaques on the tongue, palate, and buccal mucosa. These plaques can be easily removed, leaving behind an erythematous and sometimes painful surface. Patients may experience burning sensations or discomfort in the mouth and, in some cases, altered taste (dysgeusia) or dry mouth (xerostomia) [24].

It can manifest in various forms, depending on the duration of the infection (acute or chronic) and the location within the oral cavity:

- Acute Pseudomembranous Candidiasis: Known as “thrush,” it is characterized by white or yellowish plaques on the oral mucosa, composed of desquamated epithelial cells, immune cells, yeast, and *Candida* hyphae. These plaques can be easily removed, leaving a red and erosive base. It is common in neonates and immunocompromised patients (such as those with HIV/AIDS). In users of inhaled corticosteroids, this form is frequent due to local immune suppression in the oral cavity, which facilitates excessive *Candida* growth [25,26].
- Acute Erythematous Candidiasis: Known as “antibiotic sore mouth,” it occurs after the use of broad-spectrum antibiotics that reduce the normal bacterial flora, allowing *Candida* overgrowth. It presents as red, painful areas in the oral cavity and may arise spontaneously or after the loss of pseudomembranes in pseudomembranous candidiasis [27].
- Chronic Atrophic Erythematous Candidiasis: Common in individuals with HIV or denture wearers, it manifests as chronic inflammation of the palate supporting the denture, with redness and often no visible symptoms. Factors such as poorly fitted dentures or inadequate hygiene may predispose individuals to this form [28].
- Angular Cheilitis: Affects the corners of the mouth with erythema, maceration, fissures, or crusts and is associated with oropharyngeal candidiasis or denture stomatitis. It can be caused by hematinic deficiencies, warranting a blood analysis [28].
- Chronic Hyperplastic Candidiasis: Also known as candidal leukoplakia, it is characterized by raised white plaques that cannot be easily removed. It is more prevalent in middle-aged male smokers and carries an elevated risk of malignant transformation into squamous cell carcinoma [28,29].
- Median Rhomboid Glossitis: Presents as an area of atrophy and redness in the midline of the dorsal tongue. It is associated with frequent steroid inhaler use and smoking.
- Chronic Mucocutaneous Candidiasis Syndromes: These are rare immunological disorders characterized by chronic mucocutaneous infections, with a high prevalence of oral involvement and an increased risk of developing oral squamous cell carcinoma [29].

#### 3.1.1. Oropharyngeal Candidiasis in Human Immunodeficiency Virus

Oropharyngeal candidiasis is notably more prevalent and severe in individuals with compromised immune systems, especially those living with human immunodeficiency virus (HIV). *Candida* spp. are present in the oral cavity of 40–60% of healthy individuals; however, their prevalence increases significantly among immunocompromised patients, such as those with HIV infection, where it ranges from 62% to 93% [30]. In this population, the infection may serve as a clinical marker of immunosuppression and disease progression [31]. It often presents in more extensive and refractory forms, and it may coexist with esophageal candidiasis, leading to odynophagia and significant nutritional impairment. Furthermore, patients with advanced HIV infection frequently experience recurrent episodes despite antifungal therapy, underscoring the importance of both systemic management and local preventive strategies [32]. Although the pathogenesis differs from corticosteroid-induced cases, many preventive measures such as oral hygiene, denture care, and the use of probiotics may also confer benefits in these patients and will be discussed in detail later in this article. Including this group in preventive frameworks is crucial for reducing morbidity and improving oral and systemic health outcomes.

#### 3.1.2. Oropharyngeal Candidiasis in Chronic Pulmonary Diseases

Patients with chronic pulmonary diseases, such as asthma, chronic obstructive pulmonary disease, and bronchiectasis, represent a population at increased risk for oropharyngeal candidiasis due not only to frequent use of inhaled corticosteroids (ICSs) but also to underlying immunological and mucosal changes [33]. In COPD, chronic airway inflammation, frequent antibiotic use, and impaired mucociliary clearance can create an environment conducive to fungal overgrowth [34]. Asthmatic patients often exhibit altered salivary composition and reduced levels of protective antimicrobial peptides. These changes are exacerbated by high-dose ICSs, poor inhalation technique, and inadequate oral hygiene, all of which can increase local drug deposition and fungal colonization [35].

### 3.2. Diagnostic Approach and Differential Diagnosis

The diagnosis of oropharyngeal candidiasis secondary to inhaled corticosteroid use is primarily clinical, based on visual inspection of the oral cavity and identification of characteristic lesions. The most common finding is the presence of creamy white or yellowish plaques, moderately adherent to the oral mucosa, which may detach, leaving an erythematous and sometimes painful surface. These lesions are typically located on the dorsum of the tongue, the palate, the buccal mucosa, and the oropharynx [36].

Among the most frequent symptoms are burning oral pain, a persistent sensation of irritation or stinging, and an unpleasant taste in the mouth. The lesions may extend to the corners of the mouth, leading to angular cheilitis, which manifests as fissures, ulcers, or crusted radiating cracks from the labial commissures. Important risk factors to consider during evaluation include xerostomia or hyposalivation, poor oral hygiene (especially among denture wearers), malabsorption, malnutrition, and advanced malignancy. All of these factors predispose to disruption of the normal oral microbiota and increase susceptibility to fungal colonization [36].

Although the clinical diagnosis is often sufficient, additional tests may be used to confirm the etiology or rule out differential diagnoses. The first recommended diagnostic test is a superficial smear of the lesion. In cases of diagnostic uncertainty, recurrence, or resistant forms, a biopsy of the lesion, culture of a mouth rinse sample, or even upper gastrointestinal endoscopy may be considered if esophageal involvement is suspected [37].

### 3.3. Epidemiology

The prevalence and incidence of this condition vary depending on factors such as geographic region, age, and the presence of comorbidities. Although this infection can occur in both immunocompetent and immunocompromised individuals, it is significantly more common in the latter. More than 90% of patients with HIV develop oropharyngeal candidiasis at some point during the course of the disease [29]. Additionally, patients with chronic obstructive pulmonary disease (COPD) are a particularly vulnerable group. A systematic review revealed that exposure to inhaled corticosteroids nearly triples the risk of developing oropharyngeal candidiasis (RR 2.89; 95% CI: 2.36–3.55; *p* < 0.00001; I^2^ = 32%) [38].

In the United States, the prevalence of oral candidiasis among inhaled corticosteroid users ranges from 5% to 15% [39], depending on factors such as medication dosage, frequency of use, and the patient’s oral hygiene practices. The risk increases significantly with high-dose ICS use and in patients who do not rinse their mouths after inhaling the medication [40].

In Europe, studies show a prevalence similar to that in the United States, with oropharyngeal candidiasis rates among ICS users ranging from 7% to 14%. The prevalence may be higher in vulnerable populations, such as the elderly and those with chronic diseases like diabetes. In these cases, the use of ICSs, combined with other immunosuppressive factors, significantly increases the risk of developing oropharyngeal candidiasis [40,41,42].

In Latin America, epidemiological data are more limited, but some local studies suggest that the prevalence of oropharyngeal candidiasis in patients using inhaled corticosteroids may be around 10%. Differences in access to healthcare and education on oral hygiene may influence the incidence in this region [43].

### 3.4. Risk Factors and Severity

Oropharyngeal candidiasis induced by inhaled corticosteroids (ICSs) can vary in severity, ranging from mild forms to more severe and recurrent infections, depending on various factors, including corticosteroid dose, treatment duration, and the presence of patient comorbidities [44] (Table 2). One major factor contributing to severity is the dose and duration of use, with prolonged use increasing the risk. This is because ICSs can suppress local immunity in the oral cavity, facilitating *Candida* overgrowth and the progression of the infection [44].

In immunocompromised patients, oropharyngeal candidiasis is particularly severe. Patients with HIV/AIDS, diabetes, or cancer are at the highest risk. In these individuals, the infection can rapidly progress from the oral cavity to the pharynx and esophagus, potentially causing odynophagia and leading to more serious nutritional and systemic complications. In extreme cases, the infection may spread and become invasive candidiasis, a potentially life-threatening condition [45]. In the case of diabetes, chronic hyperglycemia promotes *Candida* growth, and ICS use can exacerbate this risk [45].

Severe forms, such as chronic hyperplastic candidiasis, present as elevated white lesions in the oral cavity that cannot be removed by scraping, suggesting a deeper, treatment-resistant infection. This form is associated with a higher risk of malignant transformation into squamous cell carcinoma, particularly in middle-aged smokers.

Malnutrition is a recognized risk factor for oropharyngeal candidiasis (OPC), as it can impair immune function, compromise mucosal integrity, and alter the oral microbiota. Importantly, malnutrition should be understood in a broad sense, not only as undernutrition (e.g., protein–energy deficiency or micronutrient insufficiency) but also as overnutrition, including overweight and obesity. Both extremes of nutritional imbalance can predispose individuals to OPC through mechanisms such as low-grade systemic inflammation, dysregulation of host defenses, and oral ecological shifts that favor Candida overgrowth [46,47]. Similar to immunocompromised individuals, older adults who use ICS long-term are particularly susceptible to severe oral candidiasis due to the natural decline in immunity with age and the increased likelihood of comorbidities such as diabetes or chronic pulmonary diseases [47,48]. Children, especially those with severe asthma requiring high doses of ICS, are also at risk of developing more severe forms of oropharyngeal candidiasis, which can affect their ability to eat and their overall well-being [45,46,47,48].

## 4. Non-Pharmacological Strategies

The most important factor in controlling oropharyngeal candidiasis secondary to ICS use is patient education. It is imperative to focus on reducing the exposure of the oral cavity to conditions that promote fungal overgrowth and on minimizing the local effects of inhaled corticosteroids. A significant proportion of patients use inhaled medications incorrectly [49,50], which not only exacerbates the underlying respiratory condition but also contributes to adverse effects such as oropharyngeal candidiasis due to improper deposition of the drug in the oropharynx [50].

Therefore, identifying and implementing the most effective preventive strategies is essential, not only to avoid the progression of infection but also to prevent the so-called therapeutic cascade, in which new medications are prescribed to counteract the adverse effects of the original treatment [51].

Furthermore, asthmatic patients, regardless of whether they are receiving pharmacological treatment, often present changes in stimulated salivary components, including reductions in total protein, amylase, hexosamine, salivary peroxidase, lysozyme, and secretory IgA. These alterations further exacerbate drug-induced xerostomia. In addition, there is evidence that bronchial asthma itself may be considered a risk factor for increased gingival inflammation and dental erosion, with more severe forms of asthma being directly associated with a greater risk of dental caries and periodontal disease [52].

Given this intrinsic vulnerability, maintaining strict oral hygiene becomes essential, not only to counteract the side effects of inhaled corticosteroids but also to mitigate the baseline oral health risks associated with asthma itself. Regular oral care, patient education, and preventive dental visits are thus critical components in reducing complications and preserving both oral and systemic health (Table 3).

### 4.1. Tooth Brushing

Some studies have explored the relationship between asthma and oral health, often focusing on children whose oral health is influenced by parental behaviors. Research suggests that children using inhalers may be at greater risk for oral diseases compared to their non-asthmatic peers [53]. Maintaining proper oral hygiene, including regular tooth brushing and flossing, is crucial for preserving the balance of the oral flora and preventing excessive *Candida* proliferation. This habit helps protect the oral cavity from fungal infections, especially in individuals using inhaled corticosteroids [54,55].

Furthermore, toothpaste serves as a therapeutic vehicle to improve both individual and public oral health. Its recommendation should be grounded in the best available scientific evidence rather than expert or authoritative opinion. Fluoride remains the most effective therapeutic agent in toothpaste, enhancing the benefits of mechanical toothbrushing in preventing dental caries. Its effectiveness is dependent on concentration (at least 1000 ppm fluoride) and usage frequency (at least twice per day). Concerns about dental fluorosis from toothpaste ingestion in children have been overstated, as there is no strong evidence that [4] fluoride toothpaste should be delayed until age 3 or 4, [5] low-fluoride toothpaste prevents fluorosis, or [6] fluorosis negatively impacts the quality of life in populations exposed to fluoridated water or toothpaste. Among other active ingredients, triclosan/copolymer formulations have shown efficacy in reducing dental biofilm, gingivitis, periodontitis, calculus, and halitosis. Additionally, toothpastes containing stannous fluoride have demonstrated effectiveness in reducing plaque and gingivitis [56]. Therefore, proper oral hygiene combined with scientifically supported toothpaste use represents a fundamental strategy for maintaining oral health in individuals who use ICS.

### 4.2. Care of Dental Prosthetics

Proper care of dental prosthetics is crucial not only for managing stomatitis associated with their use but also for treating all forms of oropharyngeal candidiasis. It is vital to eliminate *Candida* that may colonize dentures, as they can serve as reservoirs for the fungus. It is estimated that over 60% [57] of removable denture users harbor *Candida* on their prosthetics. These types of prosthetics restrict oxygen access and reduce adequate salivation, creating an ideal environment for fungal growth [58].

It is recommended that patients clean and disinfect their dentures daily and remove them for at least six hours each night. For effective disinfection, dentures should be soaked in chlorhexidine and then air-dried, as air exposure also contributes to *Candida* elimination. For dentures without metal components, hypochlorite may be used instead of chlorhexidine.

Additionally, it is advised to remove dentures when using antifungal mouth rinses. In cases of chronic atrophic candidiasis, soaking the dentures in chlorhexidine before reinserting them into the mouth is beneficial. For patients suffering from denture stomatitis, it is recommended to apply topical miconazole to the inner surface of the dentures before wearing them again.

In situations where patients cannot retain the antifungal mouth rinse in their mouths for long enough, it can be helpful to mix an antifungal agent with a denture liner. However, it is important to note that combining nystatin and chlorhexidine is not recommended, as both compounds inactivate each other [58].

### 4.3. Mouth Rinsing

This self-care habit is essential for maintaining oral health in patients using inhaled corticosteroids. It is crucial that patients rinse their mouths with water or a sodium bicarbonate solution after each use of the inhaler. This simple action helps eliminate corticosteroid residues that may remain in the oral cavity, thereby reducing the risk of *Candida* overgrowth [58,59]. Additionally, proper mouth rinsing and brushing not only prevent candidiasis but also reduce the likelihood of developing cavities. Studies have found that *Candida* colonization is elevated, especially in young children with cavities [59]. Children aged 3 to 13 with cavities tend to have higher *Candida* counts [60], suggesting a link between poor oral hygiene and the presence of this fungus.

Adherence to the recommendation of performing mouth rinsing after inhaled corticosteroid (ICS) administration has been shown to be variable in clinical practice. In one study, 69.5% of participants reported performing rinsing using a technique aligned with current guidelines, which include rinsing and spitting, gargling and spitting, or brushing teeth immediately after each inhaler use. These findings are consistent with previously reported adherence rates ranging from 54.4% to 86.8%. However, almost one-third of patients reported using suboptimal rinsing techniques or not rinsing at all. Suboptimal techniques included rinsing or gargling followed by swallowing, as well as simply drinking a glass of water. The literature has documented that up to 90% of patients demonstrate some deficiency in inhalation technique, which may result in inadequate disease control and decreased quality of life. Nevertheless, the evaluation of rinsing techniques has been scarcely addressed in previous research [61]. The perceived benefit of mouth rinsing after the use of ICS appears to be influenced by the type of oropharyngeal adverse effect experienced. Patients who had developed oral candidiasis or sore mouth/throat more frequently reported a positive effect from rinsing compared to those who had only experienced hoarseness or cough. A plausible explanation is that although mouth rinsing is effective at removing drug residues deposited in the oral cavity, its effect on medication that reaches the larynx is likely limited [62].

### 4.4. Use of a Spacer Device

The correct use of a spacer or valved holding chamber (VHC) helps address poor coordination between pMDI activation and inhalation. Although some synchrony remains desirable, spacers give patients more time to inhale effectively. Breath-activated pMDIs also aid coordination but lack the full benefits of a spacer. No inhaler fully prevents rapid inhalation or failure to hold breath, though some modern devices provide auditory feedback. Larger VHCs, developed in the late 1970s, additionally allow aerosol delivery through tidal breathing [63].

Spacer and VHC characteristics, such as size, valve presence, material, interface, and compatibility, affect drug delivery effectiveness. Medium- and large-volume VHCs (100–700 mL) [64] with one-way valves [65] improve inhalation flexibility and uniform aerosol delivery, though they are less portable. Spacer material matters: metal or antistatic-lined devices avoid electrostatic charge buildup, enhancing drug delivery compared to plastic ones. Facemasks are recommended for young children or those unable to use a mouthpiece, but a good seal is crucial. Some devices provide auditory feedback to guide inhalation speed or technique. Finally, compatibility between the pMDI and spacer/VHC is essential to ensure effective drug delivery into the chamber [66].

The use of a spacer device is essential to minimize medication deposition in the oral cavity. This device improves the gravitational sedimentation of the drug, ensuring that a smaller amount of residue remains in the upper airways. Not only does this optimize treatment efficacy by ensuring a larger proportion of the medication reaches the lungs, but it also significantly reduces the risk of developing oral candidiasis. Additionally, proper inhalation technique is crucial to maximize these benefits, ensuring that the medication is effectively delivered to the lungs and not retained in the oral cavity [67].

In a recent study, patients who used metered dose inhalers (MDI) with a spacer showed a lower rate of oral candidiasis compared to those who used devices like Rotacaps. These results highlight that, by using a spacer, patients have a greater opportunity to minimize the deposition of particles in the oral cavity, which helps reduce the prevalence of oropharyngeal adverse effects, such as oral candidiasis. Additionally, it was observed that the incidence of candidiasis was higher in patients over 50 years old, emphasizing the importance of adjusting inhalation technique in this vulnerable population [68].

### 4.5. Quitting Smoking

Smoking is a well-established risk factor for the development of oropharyngeal candidiasis. Several studies have shown that smokers harbor higher levels of Candida albicans compared to non-smokers, which creates a favorable environment for infection, especially when combined with the immunosuppressive effects of inhaled corticosteroids (ICS) [67,68,69]. Although there is some debate regarding the impact of tobacco on candidiasis risk, quitting smoking not only reduces this risk but also improves overall lung health, potentially allowing for a reduction in the required ICS dose. In an experimental model, it was observed that smoking-induced oxidative stress and redox dysfunction in the oral mucosa increased susceptibility to C. albicans infection [70]. Additionally, smoking negatively affected the oral mucosal defense response by negatively regulating the NLRP3 inflammasome through the Nrf2 pathway [71].

All of the above must be focused on educating patients and their families. It is essential that patients are informed about the importance of preventive measures and adherence to recommendations to avoid the development of oropharyngeal candidiasis. Education includes explaining the risks associated with ICSs and strategies to minimize them [72,73].

### 4.6. Probiotic Lozenges

Probiotics, defined as live microorganisms that, when administered in adequate amounts, confer health benefits to the host, have been studied since the late 19th century [74]. The most commonly used probiotics belong to the genera Lactobacillus and Bifidobacterium and to a lesser extent, to Saccharomyces, Bacillus, and Escherichia. In vitro and in vivo studies have demonstrated their preventive and therapeutic effects, including metabolic functions (fiber fermentation, production of short-chain fatty acids and vitamins, and cholesterol reduction), antimicrobial activities (competitive inhibition, production of bacteriocins, antitoxin effects), and immune modulation (increased IgA levels, anti-inflammatory cytokines, and regulatory T cells) [75,76,77].

Regarding Candida, certain probiotics have been shown to exhibit antifungal activity. The administration of probiotics has been found to reduce the *Candida albicans* burden and the severity of mucosal candidiasis in immunodeficient mice. Subsequent studies confirmed that strains such as *Lactobacillus rhamnosus*, *L. paracasei*, *L. reuteri*, *L. delbrueckii*, and *L. acidophilus* inhibit the growth of *Candida* spp., biofilm formation, and virulence, through the production of organic acids, hydrogen peroxide (H_2_O_2_), and bacteriocins such as fermencin SD11. Additionally, studies have reported that these probiotics reduce the *Candida* burden by modifying oral pH and through co-aggregation with fungal cells [78,79].

In experimental studies, specifically in murine models, probiotics were shown to be even more effective than nystatin in reducing oral *Candida* colonization [77]. A recent systematic review evaluated the effect of oral consumption of probiotics, prebiotics, and synbiotics on *Candida* spp. counts in oral and palatal samples. Twelve studies were included (eight randomized controlled trials and four pre-post studies), all focused on probiotics. The meta-analysis, conducted using a Bayesian random-effects model, reported a pooled odds ratio (OR) of 0.71 (95% credible interval: 0.37–1.32), suggesting a beneficial effect on oral candidiasis reduction. When considering only the randomized controlled trials, the OR was more favorable and precise: 0.53 (95% CrI: 0.27–0.93), highlighting a particularly significant effect in denture users [74].

### 4.7. Future Perspectives and Clinical Implications

The prevention of oropharyngeal candidiasis in patients using ICS continues to be a relevant clinical challenge, particularly as ICSs remain a cornerstone in the management of asthma and COPD. Future efforts must focus on optimizing the balance between the therapeutic benefits of ICS and the minimization of their local adverse effects.

One promising avenue is the development of ICS formulations specifically designed for targeted pulmonary delivery while minimizing oropharyngeal deposition. Advances in particle engineering, such as the use of extra-fine formulations or smart carriers, may enhance lung deposition efficiency and reduce the amount of drug retained in the oropharynx, thereby lowering the risk of OPC without compromising efficacy [33].

In parallel, the integration of smart inhaler technologies and digital health platforms could significantly improve disease management. These tools can provide real-time feedback on inhaler technique, track medication adherence, and potentially detect early signs of adverse events, including local side effects such as dysphonia or oral candidiasis. By enabling personalized feedback and longitudinal monitoring, these technologies hold promise for reducing complications and enhancing therapeutic outcomes [80].

Moreover, the growing understanding of host–microbiome interactions opens new preventive and therapeutic strategies. Microbiome-based interventions, including oral probiotics and targeted microbiota modulators, could serve as adjunctive therapies aimed at restoring or maintaining a healthy oral ecosystem. These approaches may offer a novel, non-pharmacologic means to counteract the dysbiosis induced by ICS, thereby reducing susceptibility to *Candida* overgrowth [81].

Taken together, these innovations represent a shift toward precision medicine in the use of inhaled therapies. Future clinical research should focus on validating these approaches in diverse populations and care settings, evaluating not only their effectiveness in oropharyngeal candidiasis prevention but also their impact on overall disease control, quality of life, and health system costs. As the field advances, multidisciplinary collaborations between pulmonologists, pharmacologists, microbiologists, and digital health experts will be essential to translate these emerging strategies into routine clinical practice.

## 5. Discussion

The occurrence of oropharyngeal candidiasis in users of inhaled corticosteroids reveals a persistent fragility in the preventive approach to respiratory care, where non-pharmacological strategies, although well known, continue to be underestimated or poorly implemented. This situation is not merely the result of isolated clinical omissions but rather a reflection of a care logic that, at times, prioritizes prescription over education and shared responsibility in care.

Non-pharmacological measures such as mouth rinsing after inhaler use, tooth brushing, the use of spacer devices, and regular review of inhalation techniques are simple, low-cost strategies with high potential impact [82]. However, their real application in patients’ daily lives is far from optimal. Although these strategies are consistently included in clinical guidelines and recommendations, their follow-up is rarely subject to systematic verification, structured monitoring, or effective patient feedback. In many cases, healthcare systems lack standardized protocols to assess adherence to these non-pharmacological measures or to reinforce their correct implementation over time. This mismatch between clinical recommendations and actual practice contributes to a fragmented approach to care. The absence of these components within a more comprehensive and continuous therapeutic strategy leaves a gap that is often paradoxically filled by the introduction of antifungal treatment interventions that, in many instances, could have been entirely preventable through rigorous and sustained attention to patient education, proper inhalation technique, and consistent oral hygiene practices. This not only reflects a missed opportunity in preventive care but also reveals systemic weaknesses in integrating low-cost, high-impact strategies into routine clinical workflows [83].

What is most problematic is not the lack of technical knowledge about these strategies but rather the structural weakness in their implementation as part of a complete therapeutic act. The inhalation technique, for example, is a central aspect of inhaled treatment, but it is often evaluated informally, without objective tools or spaces dedicated to its continuous teaching. In many cases, it is mistakenly assumed that an initial explanation is sufficient to ensure correct technique over time, ignoring that incorrect use of the device is a frequent cause of excessive oropharyngeal deposition of corticosteroids and, therefore, of opportunistic infections [84]

Moreover, post-inhalation oral hygiene, although generally recommended in clinical protocols, is not always perceived by patients as an integral and necessary component of their treatment regimen. Instead, it is often communicated as a peripheral suggestion or informal advice rather than a structured and essential part of the therapeutic plan. This framing diminishes its perceived relevance and clinical weight, leading to low adherence and inconsistent implementation. When oral hygiene is not prescribed and reinforced with the same emphasis as pharmacological measures, patients are less likely to internalize its importance. As a result, what should function as a simple and effective preventive measure becomes marginalized, reducing its protective impact and allowing preventable complications, such as oropharyngeal candidiasis, to emerge [85]. This gap between technical knowledge and patient practice points to a failure in educational processes and in the therapeutic relationship, where a deep understanding of the treatment and its impact is replaced by superficial or poorly communicated instructions.

Therefore, oropharyngeal candidiasis associated with corticosteroid use should not be analyzed solely as an infectious complication but rather as a marker of inefficiency in the implementation of non-pharmacological measures which, if rigorously applied, could prevent many cases. This directly questions the educational and follow-up model in chronic diseases and compels a rethinking of the role of health professionals beyond prescription [86].

The response, then, does not lie solely in improving antifungal agents or adjusting corticosteroid dosages but rather in addressing the structural gaps in patient education and engagement. Strengthening self-care capacities requires more than isolated instructions; it demands the integration of continuous therapeutic education programs that are context-sensitive, patient-centered, and grounded in real-life practices. A genuinely participatory approach must be adopted, one that repositions the patient not as a passive recipient of care but as an informed, proactive partner in the therapeutic process. Only through this paradigm shift can non-pharmacological strategies such as oral hygiene, inhaler technique correction, and spacer use to move from being theoretical recommendations to lived, consistent behaviors. Without such a transformation in the care model, these measures will continue to be acknowledged in guidelines yet largely neglected in clinical practice.

This review has inherent limitations due to its narrative design, such as the absence of a systematic protocol and meta-analysis, as well as the heterogeneity of the included studies and the possible exclusion of literature in other languages. However, these issues were mitigated through a comprehensive search and rigorous selection by two independent reviewers, ensuring relevant and high-quality evidence. To address these limitations, future studies should consider well-designed randomized controlled trials focusing on standardized outcome measures and including diverse populations would strengthen the evidence base, for finally conducting systematic reviews with meta-analyses to quantify the effects of non-pharmacological interventions more precisely. Among its strengths, the review stands out for its up-to-date and practical focus on non-pharmacological strategies that are easily applicable to prevent oropharyngeal candidiasis in users of inhaled corticosteroids, thereby contributing to improved clinical management.

## 6. Conclusions

Candidiasis secondary to inhaled corticosteroids is a complication that occurs in patients requiring prolonged use and is associated with the disruption of the oral microbial balance due to corticosteroid-induced immunosuppression. Prevention is the most important measure to avoid the onset of *Candida*, and strengthening education at the time ICS is prescribed is key to preventing its occurrence. However, if the infection has already developed, rapid identification of signs and symptoms is crucial for determining appropriate management. This begins with non-pharmacological measures such as oral hygiene and, if necessary, pharmacological interventions to prevent severe and recurrent complications.

## Figures and Tables

**Table 1:** Pharmacological profile of common inhaled corticosteroids [13].

ICS	Duration of Action (Hours)	Anti-Inflammatory Potency	Common Formulation
Beclometasone	Short (6–8)	Low	Aerosol, dry powder
Budesonide	Short (6–8)	Low	Aerosol, dry powder, nebulizer
Fluticasone	Long (12–24)	High	Aerosol, dry powder
Mometasone	Long (12–24)	Medium	Aerosol, dry powder
Ciclesonide	Long (24)	High	Aerosol

ICS: inhaled corticosteroids.

**Table 2:** Factors predisposing to oropharyngeal candidiasis [45,46].

Local Factor and Patient Habit	Systemic Factors and Comorbidities	Medication Use
Poor Oral Hygiene	HIV	Use of Systemic Steroids Use of Inhaled Steroids
Poor Hygiene of Dental Prosthetics	Cancer	-
Smoking	Diabetes	-
Reduced Saliva Production	Anemia	-
Presence of Cavities	Malnutrition	-
Oral Trauma Age (particularly in children and the elderly)	Neutropenia Any Condition Involving Immunosuppression	-

**Table 3:** Non-pharmacological strategies.

Strategy	Description
Patient Education	Educate patients on correct inhaler use, ICS risks, and oral hygiene to reduce fungal overgrowth and side effects.
Tooth Brushing and Toothpaste Use	Promotes oral hygiene using fluoride toothpaste (≥1000 ppm) and brushing twice daily to prevent Candida proliferation and caries.
Care of Dental Prosthetics	Daily cleaning and disinfection of dentures, removal for 6+ h/night, and antifungal treatment application to avoid fungal reservoirs.
Mouth Rinsing After ICS Use	Rinse and spit with water or bicarbonate after ICS to reduce residue and Candida colonization. Avoid swallowing rinse.
Use of a Spacer Device	Spacer improves drug delivery to the lungs, minimizes oral deposition, and reduces candidiasis, especially in older adults.
Quitting Smoking	Smoking increases Candida risk and ICS’s side effects. Cessation reduces fungal burden and improves mucosal immunity.
Probiotic Lozenges	Lactobacillus strains reduce Candida growth and biofilm formation; some studies show probiotics outperform nystatin in murine models.
Future Perspectives	Future strategies include extrafine ICS formulations, smart inhalers for feedback, and microbiome-targeted therapies.

## Data Availability

The original contributions presented in this study are included in the article. Further inquiries can be directed to the corresponding author.
